# BANSMDA: a computational model for predicting potential microbe-disease associations based on bilinear attention networks and sparse autoencoders

**DOI:** 10.3389/fgene.2025.1618472

**Published:** 2025-08-08

**Authors:** Xianzhi Liu, Mingmin Liang, Ge Yu, Shichang Tang, Ouxiang Wu, Bin Zeng, Lei Wang

**Affiliations:** ^1^ School of Information Engineering, Hunan Vocational College of Electronic and Technology, Changsha, China; ^2^ School of Intelligent Equipment, Hunan Vocational College of Electronic and Technology, Changsha, China; ^3^ School of Continuing Education, Central South University of Forestry and Technology, Changsha, China; ^4^ Big Data Innovation and Entrepreneurship Education Center of Hunan Province, Changsha University, Changsha, China

**Keywords:** computational model, microbe-disease associations, bilinear attention networks, sparse autoencoder, prediction

## Abstract

**Introduction:**

Predicting the relationship between diseases and microbes can significantly enhance disease diagnosis and treatment, while providing crucial scientific support for public health, ecological health, and drug development.

**Methods:**

In this manuscript, we introduce an innovative computational model named BANSMDA, which integrates Bilinear Attention Networks with sparse autoencoder to uncover hidden connections between microbes and diseases. In BANSMDA, we first constructed a heterogeneous microbe-disease network by integrating multiple Gaussian similarity measures for diseases and microbes, along with known microbe-disease associations. And then, we employed a BAN-based autoencoder and a sparse autoencoder module to learn node representations within this newly constructed heterogeneous network. Finally, we evaluated the prediction performance of BANSMDA using a 5-fold cross-validation framework.

**Conclusion:**

Experiments results showed that BANSMDA achieved superior performance compared to other cutting-edge methods. To further assess its effectiveness, we carried out case studies on two common diseases (including Asthma and Colorectal carcinoma) and two important microbial genera (including *Escherichia* and *Bacteroides*), and in the top 20 predicted microbes, there were 19 and 20 having been confirmed by published literature respectively. Besides, in the top 20 predicted diseases, there were 19 and 19 having been confirmed by published literature separately. Therefore, it is easy to conclude that BANSMDA can achieve satisfactory prediction ability.

## Introduction

A multitude of studies has underscored the significant influence that parasitic microbial communities within the human body exert on our metabolic processes (Kau et al., 2011). These microbes offer a range of benefits to humans, including the collection and storage of energy, the facilitation of organic compound absorption, and the defense against external microbes and diseases (Kim et al., 2018). Moreover, shifts within these microbial populations can potentially influence our health (Racanelli et al., 2018). Research also indicates that the onset of chronic diseases is intricately linked to the symbiotic microbiota that reside within us, particularly anomalies in the gut microbiota’s genome, which may lead to alterations in the human genome (Sampson et al., 2016). Furthermore, the diversity of microbial communities is closely associated with the incidence and progression of cardiovascular and neurodegenerative diseases, exerting a substantial impact on human health (Toya et al., 2020; Cryan and Dinan, 2012). Consequently, the deliberate modulation of the human microbiota’s abundance presents a promising avenue for bolstering our disease resistance and enhancing global health (Desbonnet et al., 2010). Specifically, fine-tuning the equilibrium of the gut microbiota can aid in combating viral infections. Additionally, the supplementation of lactobacilli and bifidobacteria not only assists in pain relief but also plays a role in regulating emotions and reducing anxiety, highlighting the multifaceted benefits of these microbial allies (Turnbaugh et al., 2007).

Given the inextricable links between microbes and human health, scientists have embarked on numerous microbiome-based disease research projects since the 21st century (Gilbert et al., 2010; Sun et al., 2018). However, traditional wet-lab methods for detecting microbial-disease associations, such as culture-dependent and quantitative methods, are time-consuming, requiring extensive periods for cultivation, observation, and detection of a wide variety of microbes. These methods also suffer from a degree of arbitrariness and inherent risks. To surmount the limitations of biological research, the application of computational methods has been on the rise in recent years, spurred by rapid advancements in biotechnology. Additionally, experimentally validated databases linking microbes to diseases, such as HMDAD (Ma et al., 2017) and Disbiome (Janssens et al., 2018), have been established, providing invaluable data resources for scientific inquiry. These databases serve as a treasure trove of information, facilitating a deeper understanding of the complex interplay between microorganisms and human health. For instance, reference (Park et al., 2021) employs sophisticated computational approaches, including hierarchical long short-term memory (LSTM) networks and ensemble parsing models, to unravel the complex associations between microbes and diseases. Reference (Lu et al., 2023) employs a cutting-edge combination of autoencoders and graph convolutional networks to predict potential associations between microbes and diseases. Reference (Chen et al., 2024a) introduces a pioneering human microbiota disease association prediction model that is grounded in multi-view latent feature learning, and reference (Hu et al., 2023) introduces a microbe-disease association prediction model based on generative adversarial networks.

**FIGURE 1:**
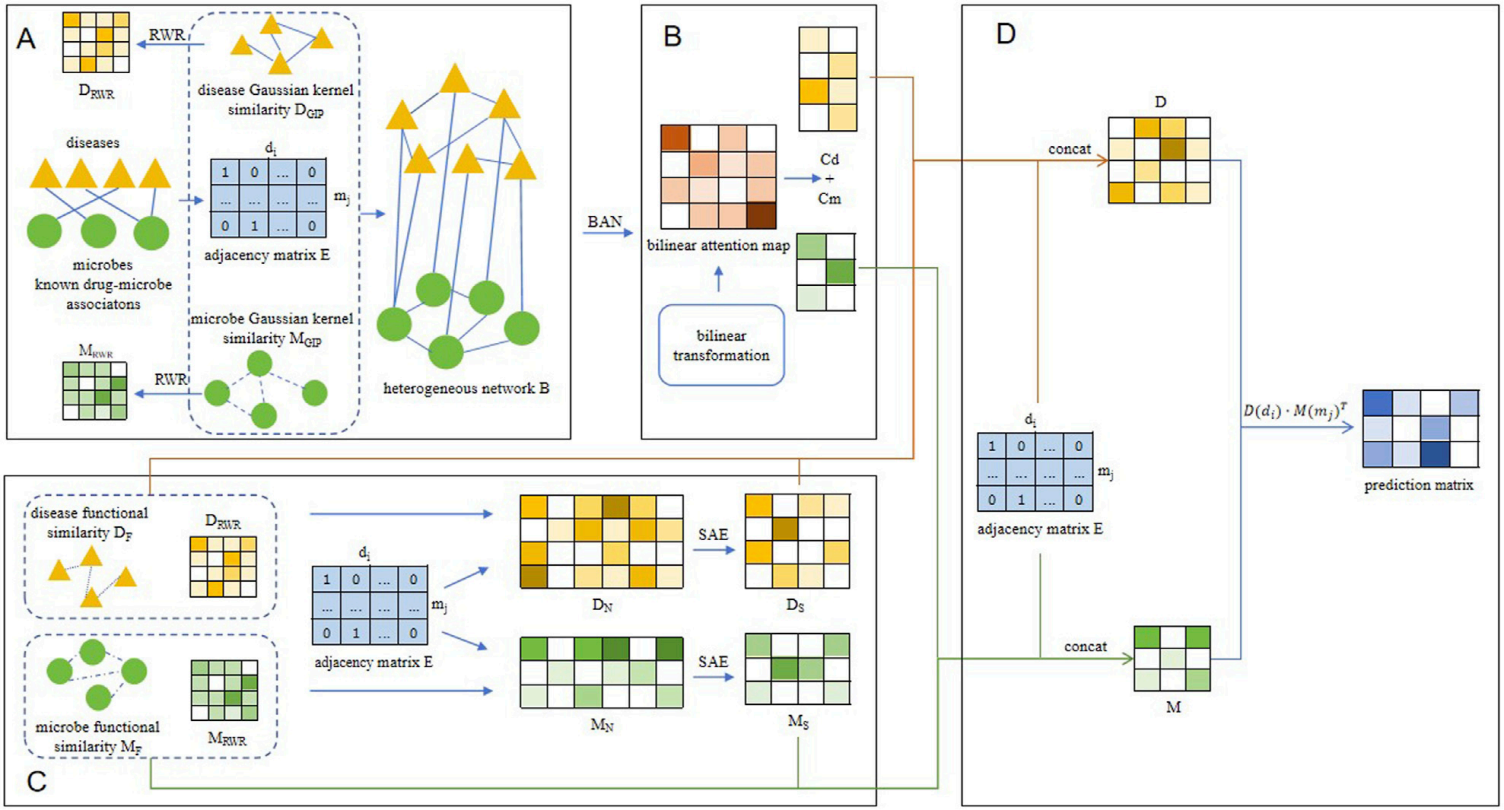
The overall structure diagram of BANSMDA. **(A)** The heterogeneous microbe-disease network was established by amalgamating the microbe Gaussian similarity network, the disease Gaussian similarity network, and known associations between microbes and diseases. **(B)** Learning node representation based on BAN. **(C)** Learning node representation based on SAE. **(D)** Predicting the final scores of potential microbe-disease associations.

In this manuscript, we proposed an innovative forecasting framework named BANSMDA to infer possible microbe-disease associations by combining Bilinear Attention Networks (BAN) with sparse autoencoder (SAE). By fusing the nuanced feature interactions discerned by BAN (Liang et al., 2025) with the proficiency of SAE in feature dimensionality reduction and representation learning, BANSMDA is expected to deliver more precise and dependable predictions within the realm of microbe-disease associations. As depicted in Figure 1, the key contributions of the BANSMDA encompass the following innovative aspects.

(1) A novel heterogeneous network *B* composed of microbes and diseases has been created by integrating the functional similarity network of microbes, the functional similarity network of diseases, and the existing microbe-disease associations.
(2) Utilize the BAN framework and the SEA framework respectively to derive node attribute representations within the heterogeneous network *B.*
(3) Integrate the attribute representations of the two types of nodes, leveraging their multiple original features, to construct comprehensive node features within network *B.*
(4) Calculate potential association scores for microbe-disease pairs using their feature matrices.

## Materials and methods

### Data sources

In this section, we would download known microbe-disease associations from public databases including HMDAD and Disbiome separately, among them, HMDAD (Ma et al., 2017) was compiled by Ma et al., in 2017, and after eliminating duplicate entries, we downloaded 450 distinct association pairs involving 39 diseases and 292 microbes. Besides, Disbiome (Janssens et al., 2018) was compiled by Janssens Y et al., in 2018, and after eliminating duplicate entries, we extracted 5,573 established associations between 240 diseases and 1,098 microbes. Detailed statistical information was shown in Table 1.

**TABLE 1:** The specific statistical data.

Dataset	Diseases	Microbes	Associations
HMDAD	39	292	450
Disbiome	240	1,098	5,573

## Methods

### Microbe-disease incidence matrix

The incidence matrix $E\in R^{n_{d}\times n_{m}}$ is a square matrix used to represent a bipartite graph where one set of vertices represents diseases ( $n_{d}$ ) and the other set represents microbes ( $n_{m}$ ). The matrix is structured such that the rows correspond to diseases and the columns correspond to microbes. Each entry $e_{ij}$ in the matrix E indicates the presence or absence of an interaction between disease $d_{i}$ and microbe $m_{j}$ . Specifically, if $E_{ij}=1$ , it means there’s a relationship between disease $d_{i}$ and microbe $m_{j}$ . If $E_{ij}=0$ , there’s no known relationship Equation 1 shows how it works:

$$E_{ij}=\left\{\begin{array}{l} 1,if\;d_{i}\;associates\;with\;m_{j}\\ 0,otherwise \end{array}\right.$$ (1)

### Microbe/Disease Gaussian kernel similarity

The similarity $D_{GIP}\left(d_{i},d_{j}\right)\in R^{n_{d}\times n_{d}}$ between diseases $d_{i}$ and $d_{j}$ , as measured by the Gaussian kernel, can be determined using Equation 2:

$$D_{GIP}=\exp\left(-{\gamma_{d}\left\|E\left(d_{i}\right)-E\left(d_{j}\right)\right\|}^{2}\right)$$ (2)

In the Gaussian kernel similarity, $\left\|E\left(d_{i}\right)-E\left(d_{j}\right)\right\|$ refers to the Euclidean distance between two diseases. The parameter $\gamma_{d}$ as shown in Equation 3, is key in controlling how the distance affects the similarity measure:

$$\gamma_{d}=1/\left(\frac{1}{n_{d}}\sum_{i=1}^{n_{d}}{\left\|E\left(d_{i}\right)\right\|}^{2}\right)$$ (3)

Absolutely, the Gaussian kernel similarity $M_{GIP}\left(m_{i},m_{j}\right)\in R^{n_{m}\times n_{m}}$ can be applied to measure the similarity between microbes as well as shown in Equations 4, 5:

$$M_{GIP}=\exp\left(-{\gamma_{m}\left\|E\left(m_{i}\right)-E\left(m_{j}\right)\right\|}^{2}\right)$$ (4)

$$\gamma_{m}=1/\left(\frac{1}{n_{m}}\sum_{i=1}^{n_{m}}{\left\|E\left(m_{i}\right)\right\|}^{2}\right)$$ (5)

### Microbe/disease functional similarity

Under the premise that diseases with similar characteristics are likely to interact with analogous genes (Wei and Liu, 2020; Xu and Li, 2006), we proceeded to calculate the functional similarity of diseases based on the functional associations among genes implicated in these diseases. The recently unveiled HumanNet v2.0 database serves as a potent tool for efficiently accessing gene interactions (Hwang et al., 2019; Long et al., 2021), with each interaction being accompanied by a log-likelihood score (LLS). This LLS quantifies the likelihood of a functional connection existing between genes. For a pair of diseases, denoted as $d_{i}$ and $d_{j}$ , we initially extracted their respective associated gene sets, denoted as $G_{i}=\left\{g_{i1},g_{i2},\ldots ,g_{im}\right\}$ and $G_{j}=\left\{g_{j1},g_{j2},\ldots ,g_{jm}\right\}$ . Here, m represents the count of genes within set $G_{i}$ , and n represents the count of genes within set $G_{j}$ . We then defined the functional association between a single gene g and a gene set $G=\left\{g_{1},g_{2},\ldots ,g_{k}\right\}$ using Equation 6:

$${PG}_{\left(g\right)}=\max\left(PSS\left(g,g_{i}\right)\right)$$ (6)

where $g_{i}\in G$ , and the functional similarity score between genes, represented by PSS , is defined as shown in Equation 7:

$$PSS\left(g,g_{i}\right)=\left\{\begin{array}{l} {LLS}^{\prime }\left(g_{i},g_{j}\right),if\;i\neq j\\ 1,if\;i=j \end{array}\right.$$ (7)

where ${LLS}^{\prime }$ represents the normalized LLS of genes, which is defined as shown in Equation 8:

$${LLS}^{\prime }\left(g_{i},g_{j}\right)=\frac{LLS\left(g_{i},g_{j}\right)-{LLS}_{\min}}{{LLS}_{\max}-{LLS}_{\min}}$$ (8)

where ${LLS}_{\max}$ and ${LLS}_{\min}$ denote the maximum and minimum LLS in the HumanNet database, respectively. Ultimately, we articulated the disease functional similarity using Equation 10:

$$D_{FUN}\left(d_{i},d_{j}\right)=\frac{\sum_{g_{t}\in G\left(d_{i}\right)}{PG}_{\left(d_{i}\right)}\left(g_{t}\right)+\sum_{g_{t}\in G\left(d_{j}\right)}{PG}_{\left(d_{j}\right)}\left(g_{t}\right)}{m+n}$$ (9)

In terms of microbe functional similarity, we employed the methodology advanced by Kamneva. (2017). To ascertain the functional similarity among microbes. To meticulously determine the functional similarity for any given pair of microbes, we initially sourced the protein-protein functional association network from the STRING v11 database (Szklarczyk et al., 2019). Utilizing the similarity scores derived therefrom, we constructed a microbe functional similarity matrix, $M_{FUN}$ , wherein each entry $M_{FUN}\left(m_{i},m_{j}\right)$ denotes the degree of similarity between microbe $m_{i}$ and microbe $m_{j}$ .

### Constructing the heterogeneous network B

By fusing the microbe-disease adjacency matrix with the disease Gaussian kernel similarity matrix and the microbe Gaussian kernel similarity matrix, as shown in Equation 10, we have crafted a heterogeneous network:

$$B=\left[\begin{array}{cc} D_{GIP} & E\\ E^{T} & M_{GIP} \end{array}\right]$$ (10)

where $E^{T}$ represents s transposition.

### BAN model

Bilinear Attention Networks (BAN), introduced by Kim in 2018, are composed of a central component known as the bilinear attention mechanism, which is designed to learn the distribution of attention by considering the bilinear interactions between input channels. This network employs two critical techniques to enhance feature interaction and manage complex data relationships: bilinear transformation and attention mechanisms. Bilinear transformation, which uses a weight matrix and an additive bias to process input features, is adept at revealing nuanced relationships within complex datasets, providing a robust framework for analyzing interactions. Its formula can be expressed as:

$$x=a^{T}Ha+b$$ (11)

In Equation 11, a represents the input vector to the BAN, H is a trainable weight matrix, b denotes the bias term, and x is the resulting output vector from the BAN. The forward propagation process of the BAN can be described as follows:

$$y_{ReLU}=ReLU\left(H_{1}x+b_{1}\right)$$ (12)

$$y=H_{2}y_{ReLU}+b_{2}$$ (13)

$$y=H_{2}ReLU\left(H_{1}x+b_{1}\right)+b_{2}$$ (14)

In Equation 12, H_1_ denotes the weight matrix from the input layer to the hidden layer, b_1_ represents the bias vector of the hidden layer, and x, defined in Equation 11, corresponds to the input vector. In Equation 13, H_2_ and b_2_ are the weight matrix from the hidden layer to the output layer and the output layer’s bias vector, respectively. By substituting Equation 12 into Equation 13, we derive the final output y and a streamlined forward propagation formula, Equation 14, which explicitly formalizes the computation process. The activation function used within the network is ReLU, as defined in formula 15, which introduces non-linearity to the model and helps in learning complex patterns. The feature vector that undergoes processing by this ReLU activation function is referred to as $y_{ReLU}$ .

$$reLU\left(z\right)=\left\{\begin{array}{l} z,z>0\\ 0,otherwise \end{array}\right.$$ (15)

By feeding the heterogeneous network B into the BAN, a low-dimensional matrix $C=\left[\begin{array}{c} C_{d}\\ C_{m} \end{array}\right]\in R^{\left(n_{d}+n_{m}\right)\times l}$ is produced, where indices $C_{d}$ and $C_{m}$ denote the disease nodes and microbial nodes, respectively. During the computation, the cross-entropy function is utilized for optimization purposes.

### SAE model

To effectively capture both the local and global topological intrinsic features of nodes, we have further implemented an enhanced version of Random Walk with Restart (RWR) on the $D_{FUN}$ . The RWR is defined as shown in Equation 16:

$$r_{i}^{l+1}=\varphi Xr_{i}^{l}+\left(1-\varphi \right)\theta_{i}$$ (16)

In Equation 16, φ denotes the restart probability. X signifies the transition probability matrix, and $\theta_{i}$ represents the initial probability vector for node i . The definition of the initial probability vector is as shown in Equation 17:

$$\theta_{ij}=\left\{\begin{array}{l} 1,if\;i=j\\ 0,otherwise \end{array}\right.$$ (17)

Following the aforementioned RWR process, it becomes evident that we can derive a new matrix $D_{RWR}$ . Similarly, after applying the improved RWR on $M_{FUN}$ , we can obtain a new matrix $M_{RWR}$ .

Consequently, by amalgamating all the matrices *E*, $D_{FUN}$ , and $D_{RWR}$ , as shown in Equation 18: we can construct a new disease matrix $D_{N}$ :

$$D_{N}=\left[E;D_{FUN};D_{RWR}\right]$$ (18)

Similarly, by integrating *E*, ${\;M}_{FUN}$ , and $M_{RWR}$ a new microbial matrix $M_{N}$ can be obtained as shown in Equation 19:

$$M_{N}=\left[E^{T};M_{FUN};M_{RWR}\right]$$ (19)

Then, use the above two matrices as inputs to the sparse autoencoder (SAE). SAE excel in feature extraction and dimensionality reduction, enabling them to distill crucial features from intricate microbial data and reduce its complexity, which is particularly valuable for managing high-dimensional biomedical data. Additionally, the incorporation of sparsity penalties within SAE helps to constrain the activation of neurons in the hidden layer, thereby enhancing the model’s feature extraction capabilities. This sparse representation not only boosts predictive accuracy but also contributes to the interpretability of the model. SAE consists of the following steps:

Encoding process: Input data *x* is converted into a hidden layer representation *h* through an encoder, and ReLU is used as a non-linear activation function after linear transformation. The specific formula is as shown in Equation 20:

$$h=ReLU\left(W_{encoder}x+b_{encoder}\right)$$ (20)

Among them, $W_{encoder}$ is the weight matrix of the encoder, and $b_{encoder}$ is the bias term.

Decoding process: The hidden layer representation *h* is reconstructed back to the original data $x^{\prime }$ through the decoder. The definition of x′ is shown in Equation 21. This process is also a linear transformation followed by a nonlinear activation function ReLU :

$$x^{\prime }=ReLU\left(W_{decoder}x+b_{decoder}\right)$$ (21)

Among them, $W_{decoder}$ is the weight matrix of the decoder, and $b_{decoder}$ is the bias term.

Refactoring loss: Refactoring error is an indicator that measures the difference between the reconstructed data $x^{\prime }$ and the original data x , represented by the binary cross entropy (BCE) loss function. The specific form is shown in Equation 22:

$$L_{recon}=BCE\left(x,x^{\prime }\right)=-\sum_{i}\left[x_{i}\log\left(x_{i}^{\prime }\right)+\left(1-x_{i}\right)\log\left(1-x_{i}^{\prime }\right)\right]$$ (22)

Sparsity loss: To introduce sparsity, SAE adds a sparsity penalty term to the loss function, which is typically based on L1 regularization. The specific form is shown in Equation 23:

$$L_{sparsity}=\lambda \sum_{j}\left|h_{j}\right|$$ (23)

where λ is the regularization coefficient and $h_{j}$ is the activation value of the hidden layer.

Total loss function: The total loss function, which is the sum of the reconstruction loss and the sparsity loss, serves as the objective function for optimization during the training process. It can be articulated as shown in Equation 24:

$$L_{total}=L_{recon}+L_{sparsity}$$ (24)

Consequently, by feeding the disease matrix $D_{N}$ and the microbe matrix $M_{N}$ into the SAE individually, we can derive matrices $D_{S}$ and $M_{S}$ , respectively.

### Microbe/disease feature matrix

Based on the processing results of BAN and SAE models, by integrating the disease matrix $C_{d}$ , $D_{S}$ , $D_{RWR}$ , $D_{FUN}$ and the adjacency matrix *E*, inspired by Xuan et al. (2020) (Long et al., 2021), we can construct a new disease feature matrix D as shown in Equation 25:

$$D=\left[C_{d};\;D_{S};\;D_{RWR};\;E;\;D_{FUN};\;E\;\right]$$ (25)

Similarly, integrating the microbial matrix $C_{m}$ , $M_{S}$ , $M_{RWR}$ , $M_{FUN}$ and the adjacency matrix *E*, we can construct a new microbe feature matrix M as shown in Equation 26:

$$M=\left[C_{m};\;M_{S};\;{E^{T};\;M}_{RWR};\;E^{T};\;D_{FUN}\;\right]$$ (26)

### Calculating the final predicted scores of potential microbe-disease associations

The dot product of two vectors serves as an effective mechanism for modeling interactions, highlighting the shared aspects of these interactions while diminishing the distinct information they might carry. Consequently, for any given disease $d_{i}$ and microbe $m_{j}$ , we can determine their predicted association scores by computing the inner product of their feature representations, as shown in Equation 27:

$$R_{ij}=Sigmoid\left(D\left(d_{i}\right)\cdot {M\left(m_{j}\right)}^{T}\right)$$ (27)

### Experiments and results

In this part, we began by conducting a sensitivity analysis of crucial parameters to improve the model’s effectiveness. Next, we chose six state-of-the-art techniques to benchmark against BANSMDA. Additionally, to confirm the model’s reliability, we selected two exemplary microbes and diseases for evaluation.

### Parameter sensitivity analysis

Considering the actual situation of the model, we identified and analyzed four parameters that have a significant impact on the final prediction results. These include the *L*2 regularization parameter $\left(\lambda \right)$ ,which is named $l_{1}$ ,in the BAN model, φ in the RWR of formula (14), the learning rate $l_{2}$ and the average activation ρ in the SEA model.

**FIGURE 2:**
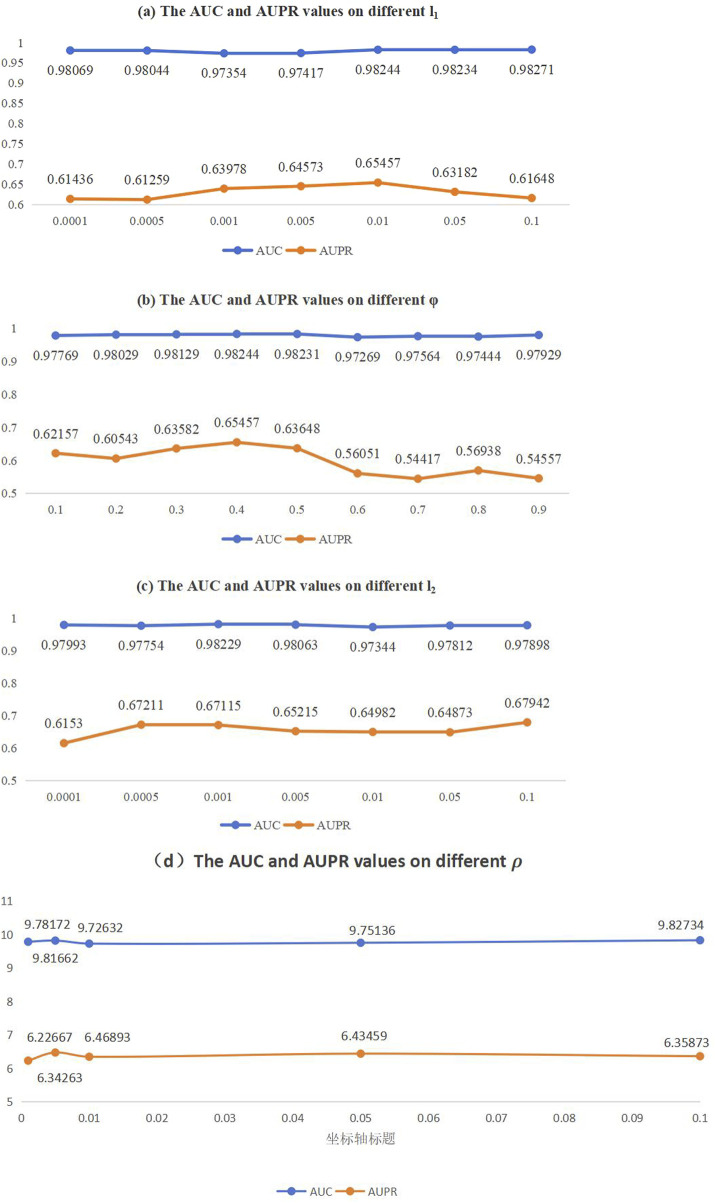
AUC and AUPR values on different parameter sensitivity analysis. **(A)** The AUC and AUPR values on different $l_{1}$ , **(B)** The AUC and AUPR values on different φ , **(C)** The AUC and AUPR values on different $l_{2}$ ,**(D)** The AUC and AUPR values on different ρ.

In this section, our objective is to determine the optimal settings while maintaining the separation of the training and testing datasets. Specifically, The range of values for $l_{1}$ is set to $\left\{0.0001,0.0005,0.001,0.005,0.01,0.05,0.1\right\}$ . The range of values for φ is $\left\{0.1,0.2,0.3,0.4,0.5,0.6,0.7,0.8,0.9\right\}$ . The range of values for $l_{2}$ is $\left\{0.0001,0.0005,0.001,0.005,0.01,0.05,0.1\right\}$ . The range of values for ρ is {0.001,0.005,0.01,0.05,0.1}.Subsequently, we employed a 5 - fold cross - validation (CV) method to evaluate the area under the receiver operating characteristic curve (AUC) and the area under the precision - recall curve for the parameter configurations. In the parameter validation experiment, we first set one parameter, e.g., *l*_2__lamda, to a fixed value. Then, in each epoch, we changed the value of one of the other parameters. After that, we collected all AUC and AUPR values under such circumstances. Finally, we calculated the average of the obtained AUC and AUPR values respectively, and used them as the final results for that particular parameter setting. In the 5-fold CV experiment, we first randomly assigned 80% of the dataset, including both identified and unidentified associations, to the training set, with the remaining 20% reserved as the independent test set. We then divided the training set into five equal-sized subsets to perform 5-fold cross-validation. Using the HMDAD dataset, we independently conducted five cross-validation runs. After the cross-validation was completed, the model’s performance was evaluated on different subsets of the training set. Finally, we used the pre-defined independent test set to assess the model’s final performance. As shown in Figure 2, the model achieves the best performance when the parameter value are configured as follows: $l_{1}=0.01,\varphi =0.4,l_{2}=0.005,\rho =0.005$ .

## Comparison with advanced methods

To further validate the predictive accuracy of BANSMDA, this section includes a comparative analysis with six prominent and competitive approaches. In the experiment, we employed the same 5-fold cross-validation technique on the HMDAD dataset for each method to ensure fair and consistent comparisons.

MOSFL-LNP (Chen et al., 2024b): This method involves preprocessing a similarity matrix, integrating low-order and high-order learning, optimizing and solving the associated equations, and finally normalizing the predicted association score matrix.

LRLSHMDA (Wang et al., 2017): This is a semi-supervised computational model utilizes the Gaussian interaction profile kernel similarity and the Laplacian regularized least squares classifier to predict the Potential Microbe-Disease associations.

BIRWMP (Shen et al., 2018): This method is a computational model based on bidirectional random walk, which predicts potential microbe-disease associations by conducting multipath analysis on microbe and disease similarity networks.

NTSHMDA (Luo and Long, 2020): A computational model based on neighborhood topology similarity, which is used for predicting the potential microbe-disease associations.

KATZHMDA (Chen et al., 2016): This is a computational method based on the KATZ algorithm, which calculates the association between microbes and diseases by considering the number and length of paths connecting two nodes in a microbe-disease heterogeneous network.

HMDA_Pred (Fan et al., 2020): This method is a novel computer model based on multi-data integration and network consistency projection, used for calculating the associations between microbes and diseases.

**FIGURE 3:**
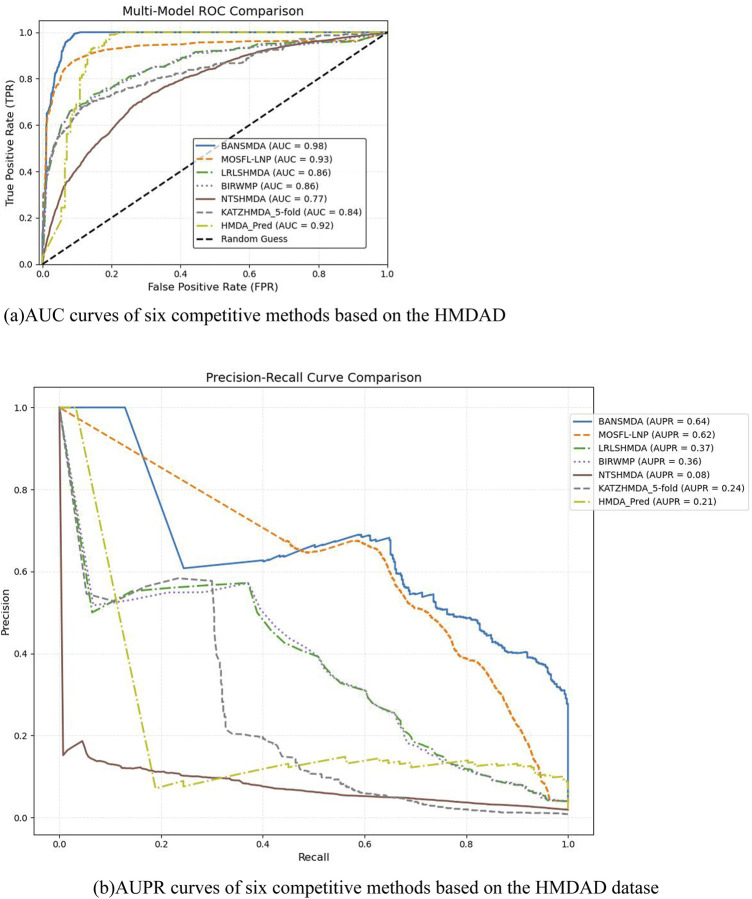
AUC and AUPR curves of six competitive methods based on the HMDAD dataset. **(A)** AUC curves of six competitive methods based on the HMDAD dataset. **(B)** AUPR curves of six competitive methods based on the HMDAD dataset.

We assessed the performance of these models under their default parameter settings and five-fold cross-validation. By leveraging the HMDAD dataset, We comprehensively evaluated our model using four key metrics: AUC, AUPR, Accuracy, and F - score, all of which were obtained through averaging over five - fold cross - validation. The results are detailed in Table 2 and visualized in Figure 3, demonstrating the superior predictive performance and accuracy of the BANSMDA model compared to other methods.

**TABLE 2:** Results of the compared methods.

Methods	AUC	AUPR	Accuracy	F1-score
BANSMDA	**0.98011 ± 0.0023**	**0.64431 ± 0.0032**	0.91245	**0.47276**
MOSFL-LNP	0.93062 ± 0.0012	0.62327 ± 0.0013	**0.91269**	0.44111
LRLSHMDA	0.85827 ± 0.0035	0.36511 ± 0.0312	0.84887	0.27597
BIRWMP	0.85669 ± 0.0015	0.36395 ± 0.0156	0.91008	0.36711
NTSHMDA	0.77131 ± 0.0020	0.0768 ± 0.0153	0.73452	0.08913
KATZHMDA	0.83502 ± 0.0034	0.23771 ± 0.0048	0.88393	0.08701
HMDA_PRED	0.91875 ± 0.0026	0.21276 ± 0.0074	0.80877	0.18082

The bold values are the maximum values of each column.

As detailed in Table 2, BANSMDA exhibits outstanding performance across three evaluation metrics: AUC, AUPR, and F1-Score. Specifically, compared to the MOSFL-LNP model, BANSMDA achieves a 5.31% improvement in AUC and a 3.37% improvement in AUPR. While slightly inferior to MOSFL-LNP in terms of Accuracy, the difference is negligible (only 0.00024). Collectively, these results confirm that BANSMDA is a highly efficient model for predicting microbe-disease associations.

**FIGURE 4:**
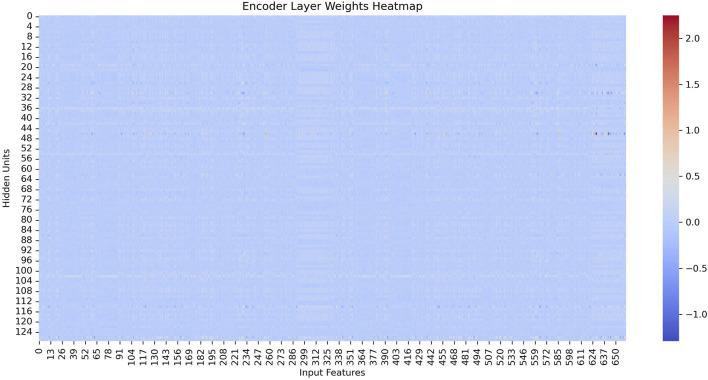
The encoder weight heat map of SAE.

As showen in Figure 4, we visualize the learned encoder weights and hidden layer activations to demonstrate the interpretability of the sparse representations. The results show that each hidden unit specializes in specific input features and exhibits sparse, selective activation patterns across samples, supporting our claim that the SAE produces interpretable representations.

Figure 4 shows the heatmap of the encoder weights after training. We observe that each hidden unit (row) assigns substantial weights to only a small subset of input features (columns), with most weights close to zero. This suggests that each hidden neuron specializes in detecting specific feature patterns in the input rather than responding indiscriminately to all dimensions. Such specialization is desirable because it allows us to attribute meaningful feature combinations to individual hidden units, enhancing interpretability of the learned representation.

**FIGURE 5:**
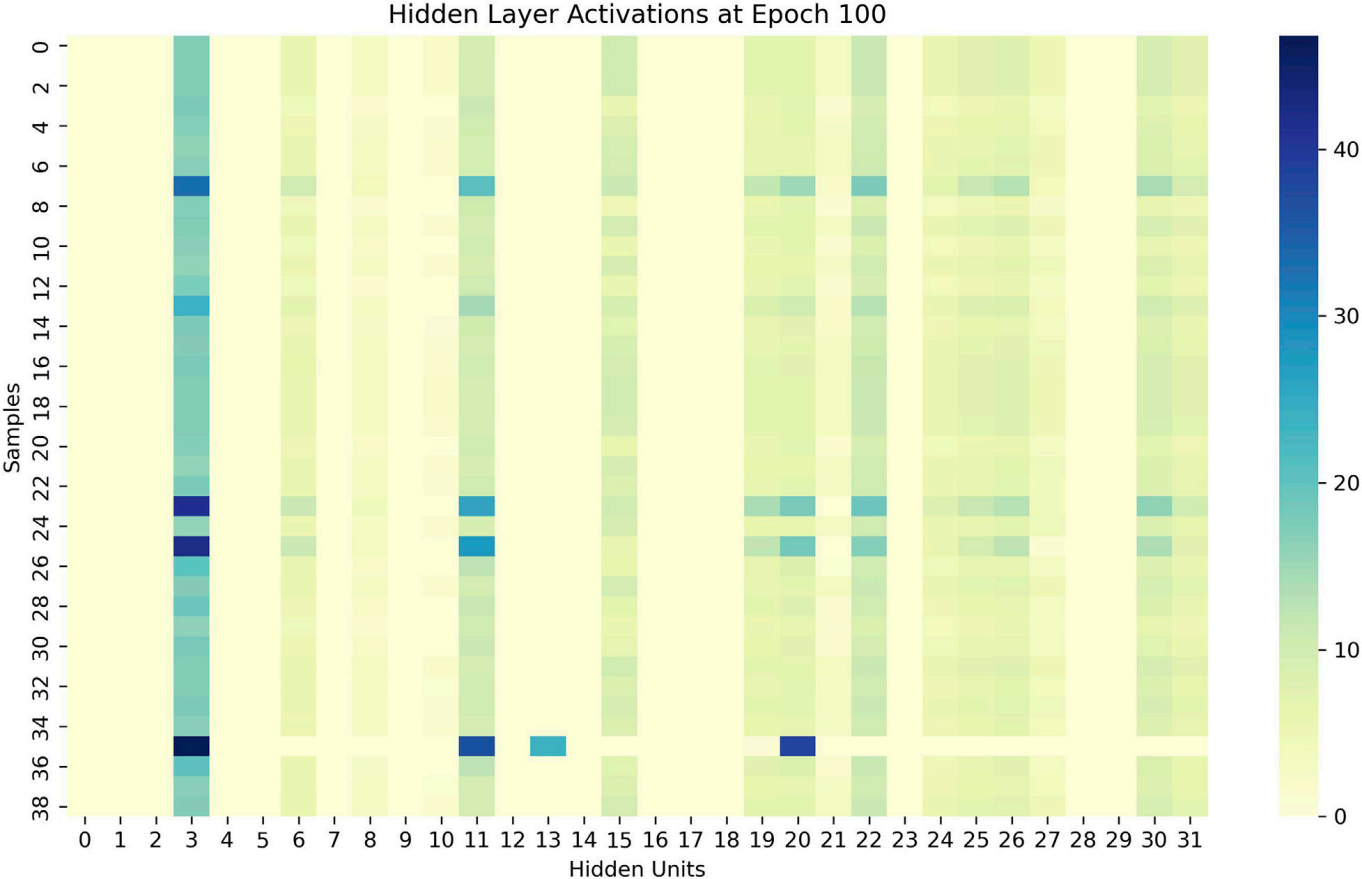
The encoder weight heat map of SAE.

Figure 5 depicts the hidden-layer activations at epoch 100. These heatmaps illustrate the activations of 32 hidden units (x-axis) across a batch of input samples (y-axis). We note two key observations: (1) the activations are sparse—for each sample, only a few hidden units exhibit high activation values, while most remain near zero; (2) the activation patterns are distinct and consistent—different hidden units activate for different samples, and the same unit responds consistently to similar samples across epochs. These findings indicate that the hidden layer learns a set of specialized, non-redundant detectors that respond selectively to specific input patterns.

### Case study

To rigorously assess the predictive performance of the BANSMDA model, we conducted case study validation using two prevalent diseases (Asthma and Colorectal carcinoma) and two clinically significant microbial genera (*Escherichia* and *Bacteroides*).

Asthma is a common chronic inflammatory disease originating in the lower airways, characterized by persistent airway inflammation (Polyxeni et al., 2021). Clinical manifestations include recurrent episodes of wheezing, coughing, chest tightness, and shortness of breath, typically exacerbated during nocturnal or early morning periods (James, 2015). A growing body of evidence from multiple references underscores a significant correlation between the pathogenesis of asthma and specific microbiota, such as *Helicobacter pylori* (Zhi et al., 2021), Proteobacteria (Kian, 2017), and Bacteroidetes (Chie, 2023). Based on the predictive scores, microbes associated with asthma were ranked in descending order according to their respective scores. As illustrated in Table 3, among the top 20 predicted microbes associated with Asthma, 19 have been confirmed by existing research indexed in PubMed.

**TABLE 3:** Top 20 Asthma-associated candidate microbes on HMDAD.

Microbe	Evidence	Microbe	Evidence
*Helicobacter pylori*	PMID:33080611	*Lactobacillus*	PMID:33882482
Proteobacteria	PMID:29161086	Burkholderia	PMID:15297563
Bacteroidetes	PMID:38155860	Faecalibacterium prausnitzii	PMID:33709404
Prevotella	PMID:28542929	Coxiellaceae	NA
*Staphylococcus*	PMID:31980492	*Clostridium*	PMID:35349868
*Haemophilus*	PMID:37287344	Clostridiales	PMID:24798552
Sphingomonadaceae	PMID:21194740	*Pseudomonas*	PMID:36167555
Comamonadaceae	PMID:27433177	Betaproteobacteria	PMID:23053501
*Clostridium difficile*	PMID:32487252	Propionibacterium	PMID:29447223
Oxalobacteraceae	PMID:21194740	Gammaproteobacteria	PMID:27889361

Notes: The top 10 microbes are listed in the first column, while the top 11–20 microbes are listed in the third column.

Colorectal carcinoma ranks as the third most common cancer globally (Chie, 2023; Inés et al., 2017). The gut microbiota is intricately involved in its development, with ecological imbalances capable of inducing colorectal carcinoma through chronic inflammatory pathways. Key bacterial taxa implicated in this multifaceted process include *Clostridium* (Hui et al., 2023), *Bacteroides* (Yasutoshi et al., 2024), and Enterobacteriaceae (Rashmi et al., 2016). As illustrated in Table 4, all of the top 20 predicted microbes associated with Colorectal carcinoma have been confirmed by existing studies in PubMed.

**TABLE 4:** Top 20 Colorectal carcinoma-associated candidate microbes on HMDAD.

Microbe	Evidence	Microbe	Evidence
Bacteroidetes	PMID:28643627	Clostridia	PMID:36941257
Firmicutes	PMID:37069401	*Haemophilus*	PMID:24725844
Prevotella	PMID:35935780	*Clostridium* coccoides	PMID:28661219
*Bacteroides*	PMID:38266708	*Fusobacterium*	PMID:26311717
Proteobacteria	PMID:27721244	*Staphylococcus*	PMID:28506660
*Helicobacter pylori*	PMID:31368293	Lachnospiraceae	PMID:36893736
*Clostridium difficile*	PMID:26691472	Enterobacteriaceae	PMID:27015276
*Staphylococcus aureus*	PMID:25495422	*Fusobacterium* nucleatum	PMID:37130518
*Lactobacillus*	PMID:35808840	*Clostridium*	PMID:36941257
Actinobacteria	PMID:27015276	Veillonella	PMID:37519587

Notes: The top 10 microbes are listed in the first column, while the top 11–20 microbes are listed in the third column.

*Escherichia* is a bacterium that embodies a dual identity, capable of functioning as both a symbiotic microbe and a pathogenic agent within the host’s body (Olivier et al., 2010). Recent research has demonstrated that specific strains of *Escherichia* are capable of causing a range of intestinal infections, including diarrhea and enteritis (James, 2005). Moreover, *Escherichia* can extend its pathogenicity beyond the gut to cause extraintestinal infections through mechanisms like fecal contamination or hematogenous dissemination (Kevin et al., 2019). As illustrated in Table 5, among the top 20 predicted diseases associated with *Escherichia*, 19 have been confirmed by existing research indexed in PubMed.

**TABLE 5:** Top 20 Escherichia-associated candidate diseases on HMDAD.

Disease	Evidence	Disease	Evidence
Type 1 diabetes	PMID:36037202	Psoriasis	PMID:33924414
Liver cirrhosis	PMID:33466521	Colorectal carcinoma	PMID:28106826
Irritable bowel syndrome (IBS)	PMID:32966000	Atopic dermatitis	PMID:36335456
Bacterial Vaginosis	PMID:38751998	Systemic inflammatory response syndrome	PMID:34997430
Periodontal	PMID:33830141	Obesity	PMID:34385401
Necrotizing Enterocolitis	PMID:37894115	Whipple’s disease	PMID:18500934
Cystic fibrosis	PMID:24178246	Kidney stones	PMID:36798915
*Clostridium difficile* infection (CDI)	PMID:36267392	Type 2 diabetes	PMID:31399369
Ileal Crohn’s disease (CD)	PMID:37800577	Guttate psoriasis	PMID:9,627,688
Crohn’s disease (CD)	PMID:36182819	Rheumatoid arthrits	NA

Notes: The top 10 diseases are listed in the first column, while the top 11–20 diseases are listed in the third column.

Bacteroidetes are significant clinical pathogens that, when they breach the intestinal barrier, can induce severe pathology. This includes bacteremia and the formation of abscesses in various parts of the body (Hannah, 2007). As illustrated in Table 6, among the top 20 predicted diseases associated with Bacteroidetes, 19 have been confirmed by existing research indexed in PubMed.

**TABLE 6:** Top 20 Bacteroidetes-associated candidate diseases on HMDAD.

Disease	Evidence	Disease	Evidence
Type 1 diabetes	PMID:34361871	Rheumatoid arthrits	NA
Liver cirrhosis	PMID:37819146	COPD	PMID:37180432
Irritable bowel syndrome (IBS)	PMID:37616338	Cystic fibrosis	PMID:38179971
Colorectal carcinoma	PMID:38266708	Crohn’s disease (CD)	PMID:35087228
Infectious colitis	PMID:36531989	Constipation Irritable bowel syndrome (IBS)	PMID:38073315
Bacterial Vaginosis	PMID:8357044	Atopic sensitisation	PMID:33741316
Necrotizing Enterocolitis	PMID:39013030	Recurrent wheeze	PMID:29600046
Periodontal	PMID:3279073	Ulcerative colitis	PMID:35087228
Type 2 diabetes	PMID:37349979	Ileal Crohn’s disease (CD)	PMID:38282618
Atopic dermatitis	PMID:33551026	*Clostridium difficile* infection (CDI)	PMID:30619112

Notes:The top 10 diseases are listed in the first column, while the top 11–20 diseases are listed in the third column.

In summary, these case studies provide additional evidence for the ability of the BANSMDA model to predict potential associations between microbes and diseases.

## Discussion

In the present research, we developed the BANSMDA model, a predictive framework combining Bilinear Attention Networks (BAN) and Sparse Autoencoders (SAE) to identify microbe-disease associations. Our model demonstrates superior performance over existing methods in capturing intricate microbe-disease relationships. However, data scarcity and excessive parameters in the BAN component may induce overfitting, potentially compromising generalization capability in real-world scenarios. Future improvements should focus on integrating biological knowledge, refining model architecture to reduce parameter redundancy, and implementing data augmentation strategies to address data limitations. Balancing model complexity against sparse datasets remains a critical challenge for practical implementation.

The significant gap between AUC-ROC and AUPR values discrepancy reflects the extreme class imbalance in HMDAD (0.5%–2% positive samples). AUPR specifically evaluates positive class identification, while AUC-ROC measures overall class discrimination. This imbalance fundamentally constrains AUPR performance, as demonstrated in prior literature. Potential solutions include rigorous negative sample validation and AUPR-optimized training objectives to enhance positive association detection.

In this study, hyperparameter validation was implemented through a combined approach of partial grid search and typical values, constrained by computational resources in this study. Specifically, given the limited sample size, the sparsity target and penalty coefficient were set to relatively low values to prevent over-regularization. Final results were obtained by averaging across multiple runs to minimize potential errors from computational limitations. Experimental results demonstrate that this approach yields hyperparameters enabling model performance approaching the theoretical optimum.

## Conclusion

In this study, we introduce a novel model called BANSMDA to predict potential associations between microorganisms and diseases. And experimental results demonstrated the superior performance of BANSMDA. It is important to highlight that data related to microbes and diseases are often characterized by sparsity. While SAE can mitigate overfitting to some extent, the substantial number of parameters introduced by BAN models may still lead to overfitting, particularly when the volume of available data is limited. This, in turn, can compromise model performance. Future research could further enhance the model’s performance by incorporating additional biological knowledge, refining the model architecture, or employing data augmentation techniques.

## Data Availability

Publicly available datasets were analyzed in this study. This data can be found here: http://www.cuilab.cn/hmdad.
